# Remifentanil-induced hyperalgesia in healthy volunteers: a systematic review and meta-analysis of randomized controlled trials

**DOI:** 10.1097/j.pain.0000000000003119

**Published:** 2023-11-30

**Authors:** Cinzia Dello Russo, Valeria Di Franco, Elisabetta Tabolacci, Natalia Cappoli, Pierluigi Navarra, Liliana Sollazzi, Francesca Rapido, Paola Aceto

**Affiliations:** a Dipartimento di Sicurezza e Bioetica, Sezione di Farmacologia, Università Cattolica Del Sacro Cuore, Fondazione Policlinico Universitario A. Gemelli IRCCS, Rome, Italy; b Department of Pharmacology & Therapeutics, Institute of Systems Molecular and Integrative Biology (ISMIB), University of Liverpool, Liverpool, United Kingdom; c Dipartimento di Scienze dell'Emergenza, Anestesiologiche e della Rianimazione, Fondazione Policlinico Universitario A. Gemelli IRCCS, Rome, Italy; d Dipartimento di Scienze della Vita e Sanità Pubblica, Sezione di Medicina Genomica, Università Cattolica del Sacro Cuore, Fondazione Policlinico Universitario A. Gemelli IRCCS, Rome, Italy; e Dipartimento di Scienze Biotecnologiche di Base, Cliniche Intensivologiche e Perioperatorie, Università Cattolica del Sacro Cuore, Rome, Italy; f Department of Anesthesia & Critical Care Medicine, Gui de Chauliac Montpellier University Hospital, Montpellier, France; g Institute of Functional Genomics, Unité Mixtes de Recherche (UMR) 5203 Centre National de la Recherche Scientifique (CNRS)-Unité 1191 INSERM, University of Montpellier, Montpellier, France

**Keywords:** Remifentanil, Pain intensity, Hyperalgesia, Withdrawal, Quantitative sensory testing, Electrical stimulation, Allodynia

## Abstract

Recent literature suggests that the withdrawal of remifentanil (RF) infusion can be associated with hyperalgesia in clinical and nonclinical settings. We performed a systematic review and a meta-analysis of randomized controlled trials with cross-over design, to assess the effect of discontinuing RF infusion on pain intensity and areas of hyperalgesia and allodynia in healthy volunteers. Nine studies were included. The intervention treatment consisted in RF infusion that was compared with placebo (saline solution). The primary outcome was pain intensity assessment at 30 ± 15 minutes after RF or placebo discontinuation, assessed by any pain scale and using any quantitative sensory testing. Moreover, postwithdrawal pain scores were compared with baseline scores in each treatment. Secondary outcomes included the areas (% of basal values) of hyperalgesia and allodynia. Subjects during RF treatment reported higher pain scores after discontinuation than during treatment with placebo [standardized mean difference (SMD): 0.50, 95% confidence interval (CI): 0.03-0.97; *P* = 0.04, I^2^ = 71%]. A significant decrease in pain scores, compared with baseline values, was found in the placebo treatment (SMD: −0.87, 95% CI: −1.61 to −0.13; *P* = 0.02, I^2^ = 87%), but not in the RF treatment (SMD: −0.28, 95% CI: −1.18 to 0.62; *P* = 0.54, I^2^ = 91%). The area of hyperalgesia was larger after RF withdrawal (SMD: 0.55; 95% CI: 0.27-0.84; *P* = 0.001; I^2^ = 0%). The area of allodynia did not vary between treatments. These findings suggest that the withdrawal of RF induces a mild but nonclinically relevant degree of hyperalgesia in HVs, likely linked to a reduced pain threshold.

## 1. Introduction

Remifentanil (RF) is a widely used short-acting opioid agonist because of its pharmacodynamic and pharmacokinetic properties.^1,23^ The lack of accumulation with prolonged infusions provides hemodynamic stability during surgery and a low risk of respiratory depression and/or delayed awakening after withdrawal.^1,2^ However, intraoperative RF use may be limited by the occurrence of opioid-induced hyperalgesia (OIH).^28,35,41,52^ To the best of our knowledge, 4 meta-analyses of randomized control trials (RCTs) have been conducted in surgical settings.^21,22,33,51^ In the meta-analysis by Fletcher and Martinez,^21^ the authors concluded that administering high doses of RF during surgery is associated with a clinically small but statistically significant increase in pain perception. The other meta-analyses explored the effectiveness of NMDA receptor antagonists or ketamine in reducing pain intensity scores and morphine consumption after RF-based general anesthesia.^22,33,51^ Two studies showed that ketamine significantly reduced postoperative pain and total morphine consumption,^22,33^ whereas another study did not reveal significant evidence that NMDA antagonists prevented RF-induced hyperalgesia.^51^ However, it has been recently emphasized that, in clinical settings, OIH assessment is challenging, due to the lack of standardized approaches to its diagnosis. Therefore, the incidence and prevalence of OIH remain unclear and may indeed be low.^9^ Although studies in healthy volunteers (HVs) do not mirror clinical pain, especially when artificial stimuli are used, they allow for consistently measuring the areas of hyperalgesia and allodynia. Therefore, in this study, we sought to determine whether there is evidence supporting the occurrence of OIH after RF withdrawal in HVs, considering that pain assessment and hyperalgesia in human experimental models outside the surgical setting have not been systematically investigated yet.

The primary aim of this study was to evaluate the impact of RF infusion withdrawal on increasing pain intensity, assessed through any scale (eg, Numerical Rating Scale and Visual Analogue Scale) using quantitative sensory testing (QST) modality (eg, electrical and thermal) in HVs. The magnitude of hyperalgesia was quantified by calculating the average difference in postinfusion pain scores between RF and placebo treatments. The secondary aims were to evaluate whether the withdrawal of RF infusion could expand the areas of hyperalgesia and allodynia compared with placebo.

## 2. Methods

### 2.1. Search strategy

**Figure 1.**
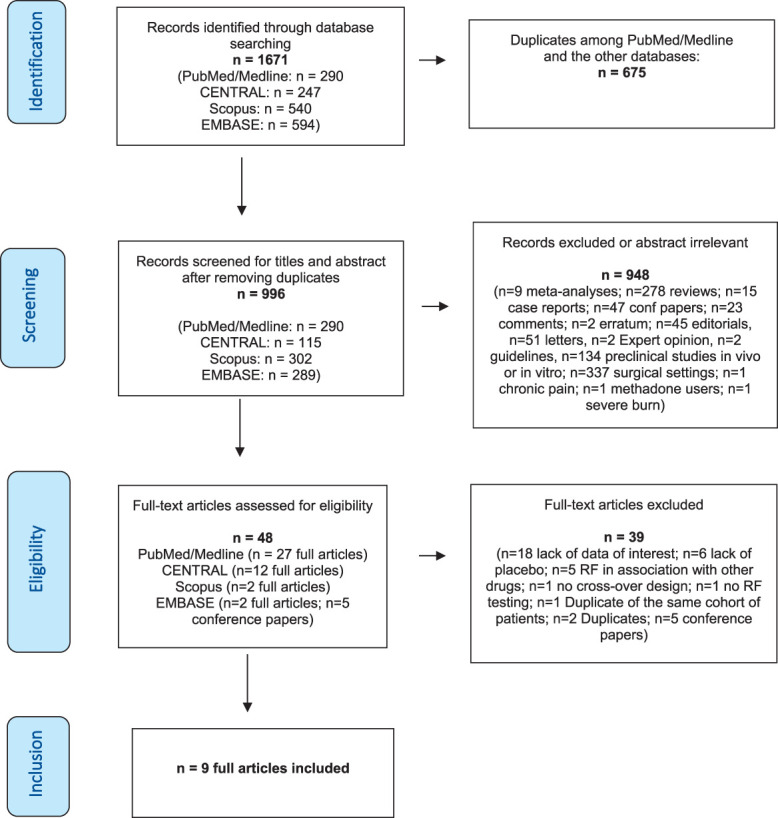
PRISMA flow diagram of the study selection process. PRISMA, Preferred Reporting Items for Systematic Reviews and Meta-Analyses.

This systematic review and the meta-analysis were performed in accordance with a registered protocol on PROSPERO (ID: CRD42022345693) and reported following the Preferred Reporting Items for Systematic Reviews and Meta-Analyses (PRISMA) guidelines.^37^ The Cochrane Central Library, PubMed/MEDLINE, Embase, and Scopus were searched up to January 27, 2023 (Fig. 1). Supplementary retrieval of additional studies and upcoming trials was also performed in Google Scholar and ClinicalTrials.gov, respectively. The search strategy, which also included MeSH terms, was based on the following search components: “remifentanil” and “hyperalgesia” [refer to search strategy details in Appendix 1 in the Online Supplementary Content (OSC), available at http://links.lww.com/PAIN/B959]. In addition, reference lists from eligible trials and related reviews were searched for additional citations that met our inclusion criteria. Studies published from the date of database inception to the last update were sought. Neither date nor language restrictions were applied. As detailed in the registered protocol, by the time of submission, the following stages had already been accomplished according to PROSPERO policy: preliminary searches, piloting of the study selection process, and formal screening of search results against eligibility criteria.

### 2.2. Study selection

All randomized controlled trials (RCTs), with cross-over design, comparing pain intensity after withdrawal of RF vs placebo infusion in HVs were considered eligible for inclusion in the systematic review and meta-analysis. In a randomized cross-over trial, each participant undergoes all treatments, serving as their own control. Washout periods between different treatments are planned to avoid a carryover effect. The “randomized” term, in the context of cross-over studies, refers to the randomly assigned order of treatments for each participant, mitigating potential biases. We excluded studies that did not compare RF vs placebo (control); studies that examined the effects of RF combined with other drugs; and studies on the pediatric population, review articles, observational studies, editorials or letters, comments, case reports/case series, and preclinical studies (both in vivo and in vitro). Titles or abstracts of studies retrieved using the search strategy and those from additional sources were screened independently by 2 reviewers (C.D.R. and V.D.F.) to identify those studies that could potentially meet the inclusion criteria. The full texts of potentially eligible studies were then retrieved and independently assessed for eligibility by 2 review team members (C.D.R. and V.D.F.). Any disagreement between them over the eligibility of specific studies was resolved by discussion, with the involvement of a third review author (P.A. or F.R.) when necessary.

### 2.3. Data extraction and quality assessment

A standardized form was used to extract data from the included studies. The following data were extracted for each study: bibliographic details such as author and year of publication, as well as data on specific study characteristics such as the first author, year of publication, country, study design, sample size (n), age (years), weight (kg), height (cm), male sex (n,%), RF infusion description, QST modality, type of scale used for pain assessment, primary outcome, and secondary outcomes ( Tables 1 and 2).

**Table 1:** Main characteristics of the studies selected for the metanalysis, including study design, experimental model, and patients' demographics.

First author (year of publication)	Country (ethics committee approval)	Study design	Control group	Remifentanil group (duration of i.v. infusion and dose)	Human experimental pain model	Mean current, mA ± SD	Subjects, n	Males, n (%)	Mean age, years ± SD	Mean weight, kg ± SD	Mean height, cm ± SD
Petersen (2001)	USA	Randomized cross-over Double-blind	Saline	40 min: 5 min at 0.05 µg/kg/min + 35 min at 0.10 µg/kg/min	Long thermal stimulation (LTS, healthy skin)/heat-capsaicin sensitization model	None	14*	9 (64.3)	34 (range, 22-56)	NR	NR
Angst (2003)	Germany	Randomized cross-over Double-blind	Saline	90 min at 0.1 µg/kg/min	Heat pain test (healthy skin)/intradermal electrical stimulation	50 ± 15	10	10 (100)	28 (range, 20-35)	77 (range, 68-97)	180 (range, 169-190)
Koppert (2003)	Germany	Randomized cross-over Double-blind	Saline	30 min at 0.05 or 0.1 µg/kg/min	Intradermal electrical stimulation	67.3 ± 16.8 (during the first 15 min)	13	13 (100)	31.2 ± 5.3 (range, 20-40)	NR	NR
Tröster (2006)	Germany	Randomized cross-over Double-blind	Saline	30 min at 0.1 µg/kg/min	Intradermal electrical stimulation	32.1 ± 12 (during the first 15 min)	15	15 (100)	29 ± 8 (range, 20-45)	76 ± 10	181 ± 8
Singler (2007)	Germany	Randomized cross-over Double-blind	Saline	30 min at 0.05 µg/kg/min	Intradermal electrical stimulation	38 ± 14 (during the first 15 min)	15	15 (100)	27 ± 4 (range, 22-35)	74 ± 10	178 ± 7
Chu (2011)	USA	Randomized cross-over Double-blind	Saline	90 min at 3 ng/mL by computer-controlled infusion	Intradermal electrical stimulation	NA	9†	10 (100)	30.3 ± 8.8 (range, 22-45)	78.3 ± 11.8	177.9 ± 4.5
Lenz (2011)	Norway	Randomized cross-over Double-blind	Saline	30 min at 1.0 ng/mL for 2 min + 2.5 ng/mL for 28 min via TCI‡	Intradermal electrical stimulation	31.2 ± 5.3 (SEM) (during the first 15 min)	16	16 (100)	29 ± 2 (SEM)	83 ± 3.4 (SEM)	183 ± 1.8 (SEM)
Comelon (2016)§	Norway	Randomized cross-over Double-blind	Saline§	30 min at 2.5 ng/mL target dose	Heat pain test/cold pressor test	None	16‖	16 (100)	30 (range, 18-40)	79 (range, 54-100)	184 (range, 165-199)
Ander (2018)¶	Sweden	Randomized cross-over Double-blind	Saline	30 min at 0.2 µg/kg/min	Cold pressor test	None	14	11 (78.6)	23 ± 3	75 ± 14	178 ± 9

The total number of subjects analyzed in these studies was 122.

* In this study, 16 subjects were originally enrolled. However, 2 patients did not complete the study sessions, after the first infusion. One volunteer withdrew consent and the other for family reasons. Demographic data refer to 14 subjects that completed the study.

† In this study, only 9 patients completed both sessions. Demographic data refer to 10 subjects screened for the study.

‡ For a 70 kg man the total dose of remifentanil administered was as it follows: 18 μg in 2 minutes, 52 μg in 3 minutes, and 212 μg in 25 minutes. The authors stated that “*by the end of the test period, that is, between 25 and 30 minutes after start, the typical infusion rate was 0.09 μg/kg/min for an effect target of 2.5 ng/mL*.”

§ This study is registered in www.clinicaltrials.gov (ID: NCT 01702389) and EudraCT (ref: 2011-002734-39), sponsored by Oslo University Hospital. However, no results were posted in these databases (databases accessed on April 17, 2023). The main objective of the study was to evaluate the effect of a gradual withdrawal of remifentanil vs the abrupt discontinuation of the drug on the development of hyperalgesia in healthy volunteers. Gradual withdrawal was obtained by 0.6 ng/mL dose reductions every 5 minutes over 15 minutes. Only one set of data is reported in the article for the placebo group, despite both gradual and abrupt withdrawals of saline were performed.

‖ In this study, 19 subjects were originally enrolled, although 3 were excluded due to the following reasons: side-effects (n = 1), data error (n = 1), and compliance problems (n = 1). Demographic data refer to 16 subjects that completed the study.

¶ This study is registered in EudraCT (ref: 2011-005780-24), although we could not find it in the database, accessed on April 17, 2023.

CG, control group; NR, not reported; RF, remifentanil; TCI, effect site target–controlled infusion.

**Table 2:** Main results from the studies selected for the meta-analysis.

First author (year of publication)	Outcome evaluation (min after RF discontinuation)	PAIN assessment scale	Pain assessment on healthy skin (yes/no)	Primary outcome: pain intensity (mean values ± SD)	Hyperalgesia assessment	Secondary outcome: area of secondary hyperalgesia (mean values ± SD)	Allodynia assessment	Secondary outcome: area of secondary allodynia (mean values ± SD)
Petersen (2001)	30	VAS (0-100)	Yes	Baseline Both: 28 ± 12 After discontinuation* CG: 25.8 ± 8.4 RF: 28.8 ± 10.5	von Frey hair stimulation (20.9 g)	Baseline (cm^2^) CG: 132 ± 71 RF: 125 ± 59 After discontinuation (% of baseline) CG: 82 ± 23 RF: 101 ± 29	Brush stimulation	Baseline (cm^2^) CG: 122 ± 54 RF: 103 ± 46 After discontinuation (% of baseline) CG: 75 ± 27 RF: 96 ± 39
Angst MS (2003)	30	VAS (0-100)	Yes	Baseline Both: CG: 47 ± 5 RF: 49 ± 11 After discontinuation CG: 47 ± 6 RF: 42 ± 8	Punctuate probe (force 160 mN; 16.3 g)	Baseline (cm^2^) CG: 44 ± 29 RF: 28 ± 15 After discontinuation (cm^2^,% of baseline)† CG: 38 ± 24 (86.36 ± 54.54) RF: 62 ± 61 (221.43 ± 217.85)	Not performed	Not performed
Koppert W (2003)	30	NRS (0-10)	No	Baseline‡§ Both: 5.5 ± 1.8 After discontinuation‡§ CG: 3.6 ± 1.8 RF (0.05 µg/kg/min): 4.7 ± 1.08 RF (0.1 µg/kg/min): 5.4 ± 1.44	von Frey hair stimulation (force 450 mN)	Baseline (cm^2^)‡§ CG: 36.7 ± 27.76 RF (0.05 µg/kg/min): 36.7 ± 27.76 RF (0.1 µg/kg/min): 39.5 ± 38.22 After discontinuation (cm^2^,% of baseline)‡§ CG: 46.75 ± 19.54; 127.38 ± 53.25 RF (0.05 µg/kg/min): 43.8 ± 44.56 RF (0.1 µg/kg/min): 64.05 ± 35.4; 162.15 ± 89.63	Cotton-wool tip	Baseline‡§ CG: 29.05 ± 27.47 RF (0.05 µg/kg/min): 29.05 ± 27.47 RF (0.1 µg/kg/min): 33.33 ± 34.40 After discontinuation (cm^2^,% of baseline)‡§ CG: 30 ± 27.40; 103.27 ± 94.32 RF (0.05 µg/kg/min): 28.1 ± 27.40 RF (0.1 µg/kg/min): 36.2 ± 34.61; 108.61 ± 103.83
Tröster A (2006)	30	NRS (0-10)	No	Baseline‡§ CG: 5.4 ± 1.55 RF: 5.6 ± 1.16 After discontinuation‡§ CG: 4.4 ± 1.16 RF: 4.8 ± 1.16	von Frey hair stimulation (force 256 mN)	Baseline (cm^2^) CG: 33.8 (range 11.2-49.0) RF: 18.8 (range 13.6-37.1) After discontinuation‡§ (% of baseline) CG: 101.23 ± 180.09 RF: 133.53 ± 194.04	Not performed	Not performed
Singler B (2007)	30	NRS (0-10)	No	Baseline‡§ CG: 5.5 ± 0.2 RF: 5.4 ± 0.5 After discontinuation‡§ CG: 4.7 ± 0.4 RF: 5.6 ± 0.5	von Frey hair stimulation (force 450 mN)	Baseline (cm^2^) Both: 32 ± 58.09 After discontinuation (% of baseline)‡§ CG: 100.1 ± 25.52 RF: 105.77 ± 47.63	Not performed	Not performed
Chu LF (2011)	30	VAS (0-10)	No	Baseline‡ CG: 3.8 ± 1.3 RF: 2.3 ± 1.1 After discontinuation‡ CG: 2.6 ± 1.6 RF: 2.8 ± 1.5	Punctuate probe (force 160 mN; 16.3 g)	Baseline (cm^2^) Both: NR After discontinuation (% of baseline)‡ CG: 100.58 ± 10.34 RF:123.86 ± 20.69	Not performed	Not performed
Lenz H (2011)	30	NRS (0-10)	No	Baseline‡§ CG: 5.2 ± 0.4 RF: 5.4 ± 0.2 After discontinuation‡§ CG: 4.24 ± 0.2 RF: 4.4 ± 0.3	von Frey hair stimulation (26 g)	Baseline (cm^2^)‡§‖: CG: 44.1 ± 20.8 RF: 50.1 ± 17.6 After discontinuation (% of baseline)‡§ CG: 93.4 ± 47.76 RF: 143.71 ± 88.08	Not performed	Not performed
Comelon M (2016)	45	NRS (0-10)	No	HPT baseline‡ Placebo: 2.71 ± 0.5 Gradual: 2.82 ± 0.5 Abrupt: 2.66 ± 0.5 After discontinuation‡ CG: 2.92 ± 0.5 Gradual RF: 2.91 ± 0.5 Abrupt RF: 3.4 ± 0.5	Not performed	Not performed	Not performed	Not performed
	50	NRS (0-10)	No	CPT¶ Baseline‡ CG: 4.56 ± 0.5 Gradual RF: 4.6 ± 0.5 Abrupt RF: 4.55 ± 0.5 After discontinuation‡ CG: 4.58 ± 0.5 Gradual: 5.05 ± 0.55 Abrupt: 5.27 ± 0.5				
Ander F (2018)	20	NRS (0-10)	No	Baseline CG: 8.6 ± 1.3 RF: 8.2 ± 1.5 After discontinuation CG: 8.6 ± 1.3 RF: 8.4 ± 1.5	Not performed	Not performed	Not performed	Not performed

* Absolute pain values were calculated from the % of baseline provided in the text and the SD was obtained through the graphs, using the following software: https://plotdigitizer.sourceforge.net.

† % of baseline was manually calculated from the values of the area in cm^2^ reported in the table.

‡ Values were obtained through the graphs, using the following software: https://plotdigitizer.sourceforge.net.

§ Standard deviation (SD) was calculated trough Review Manager software (RevMan Version 5.4) from the standard error of the mean (SEM) reported either in the text or in the graph.

‖ Data are mean values between 15 to 30 minutes of electrical stimulation.

¶ Data were not used for the meta-analysis since the evaluation was performed 50 minutes after remifentanil withdrawal and therefore outside the limits of the inclusion criteria.

CG, control group; CPT, cold pressure test; HPT, heat pressure test; NRS, Numerical Rating Scale; RF, remifentanil; VAS, Visual Analog Scale.

Two review authors (C.D.R. and V.D.F.) independently extracted data. Discrepancies were identified and resolved through discussion, involving a third author when necessary. Original investigators were contacted to request missing data, if necessary. If the authors did not reply, one additional request was sent. Back calculation of the necessary data was allowed and performed using Plot Digitizer (https://plotdigitizer.sourceforge.net).

Quality assessment of the included studies was performed using Version 2 of the Cochrane risk-of-bias tool for randomized trials (RoB 2).^44^ The certainty of evidence (Grading of Recommendations, Assessment, Development, and Evaluations [GRADE]) was conducted by 2 authors independently (C.D.R. and V.D.F.). Several domains, namely, the risk of bias, inconsistency, imprecision, indirectness, and publication bias, were assessed for all included outcomes. Any conflicts were discussed with a third author (P.A. or F.R.).

### 2.4. Preplanned group analysis and sensitivity analyses

Studies using low and intermediate doses of RF infusion and studies using electrical stimulation were analysed separately. Sensitivity analyses were performed to evaluate the possible confounding effect of sex and duration of infusion greater than 30 minutes. An additional sensitivity analysis was performed for studies using controlled infusion (CI) vs target-controlled infusion (TCI).

### 2.5. Data synthesis and meta-analysis

We compared the pain intensity (primary outcome) assessed by any pain scale, eg, Numerical Rating Scale (NRS) and Visual Analogue Scale (VAS), at 30 ± 15 minutes after discontinuation of the infusion between RF and placebo treatments. If more than one dose was used in the same study, we considered the higher one. To quantify the magnitude of the RF-induced analgesic hypersensitivity effect, the primary outcome was also described by comparing postwithdrawal vs baseline pain scores in each treatment (placebo and RF). For these analyses, we used standardized mean difference (SMD) instead of weighted average to further describe the primary outcome by comparing postwithdrawal vs basal pain scores in the single treatments (placebo and RF) to adjust values for baseline differences (please refer to the postprotocol change in the updated version at Prospero Website). The SMD expresses the intervention effect in standard units rather than the original units of measurement. In this study, the SMD allowed us to combine results from studies that used different pain scales, ensuring a more comprehensive analysis. However, as overall SMD depends on both effect size and standard deviation of the outcomes, its reading is difficult to interpret. Normally, an SMD < 0.4 indicates small effect size, an SMD 0.4 to 0.7 represents a moderate effect size, and an SMD > 0.7 means large effect size. On the other hand, calculation of weighted (on n participants) average of pain scores would have provided separate estimates for each treatment at the 2 time points. Despite being easier to interpret, this method only gives a rough indication of the effect size, does not take into account the spread of data, and needs data conversion.

We compared the areas of hyperalgesia and allodynia (expressed as % of basal value) between the 2 treatments (secondary outcomes). Standardized mean differences with the associated 95% confidence interval (CI) were calculated. Data were combined using a random-effect model with the Mantel Haenszel method. Heterogeneity was described as the I^2^ test. Data were analysed using Review Manager software (RevMan Version 5.4; the Nordic Cochrane Centre, Copenhagen: the Cochrane Collaboration, 2020) and STATA 16.1 (StataCorp. 2019. Stata Statistical Software: Release 16. College Station, TX: StataCorp LLC).

Publication bias was measured by funnel plots if enough studies (at least 10) were available, as test power is usually too low to distinguish chance from real asymmetry.^37^ The level of significance was set at *P* ≤ 0.05.

## 3. Results

### 3.1. Studies selection

Based on the initial search results, 996 titles and abstracts were examined after duplicate removal (Fig. 1). As detailed in the figure, 948 reports were rejected because they did not meet the inclusion criteria or were irrelevant to our study focus. Thirty-nine of the remaining 48 full-text articles assessed for eligibility did not meet the inclusion criteria, and 2 were duplicates. Checking reference lists of identified articles and documents did not produce any additional results. Consequently, 9 studies were included in the systematic review and were used for the quantitative analysis.

### 3.2. Study characteristics

The main characteristics of the studies are shown in Table 1. The studies selected for the systematic review (n = 9) involved 122 participants and were conducted with a randomized, double-blind, and placebo-controlled design. The mean age was 30.2 ± 1.82 (SD) years, and the total number of female patients was 8, representing 6.6% of the population. Pain intensity was evaluated on healthy skin in 2 studies, whereas it was performed at 30 minutes after RF discontinuation in 7 studies (Table 2). Table 2 provides a summary of the data extracted from the selected studies. Pain intensity was evaluated using NRS (0-10) in 6 studies, whereas 3 studies used VAS ranging between 0 and 100 mm or 0 to 10 cm.

### 3.3. Primary outcome

**Figure 2.**
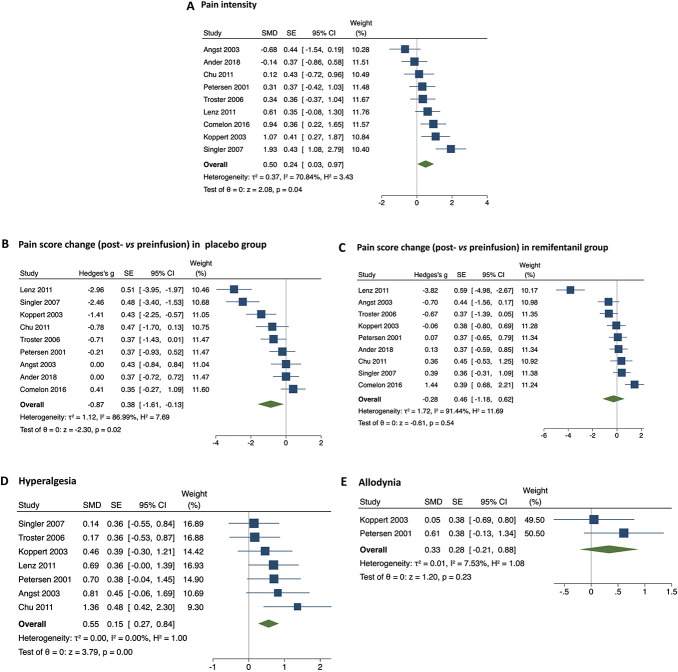
Forest plots reporting pain intensity after remifentanil withdrawal (A), pain score change (postinfusion vs preinfusion) in placebo treatment (B), pain score change (postinfusion vs preinfusion) in remifentanil treatment (C), area (expressed as % of basal value) of hyperalgesia (D), and allodynia (E) after remifentanil withdrawal. The effect size is calculated as standardized mean difference (SMD) and corresponding 95% confidence interval (95% CI). Data suggest higher pain score in RF treatment (A) and stability of pain intensity in the postinfusion vs preinfusion analysis in the RF treatment (C). RF, remifentanil.

Remifentanil withdrawal produced significantly higher pain scores than placebo discontinuation (SMD: 0.50, 95% CI: 0.03-0.97; *P* = 0.04, I^2^ = 71%) (Fig. 2A). Pre–post analyses revealed a significant decrease in pain scores only in the placebo treatment (SMD: −0.87, 95% CI: −1.61 to −0.13; *P* = 0.02, I^2^ = 87%) (Fig. 2B), whereas no significant changes were detected in the RF treatment (SMD: −0.28, 95% CI: −1.18 to 0.62; *P* = 0.54, I^2^ = 91%) (Fig. 2C).

### 3.4. Secondary outcomes

The area of hyperalgesia (expressed as % of basal value) was more extended after RF compared with placebo withdrawal (SMD: 0.55; 95% CI: 0.27-0.84; *P* = 0.001; I^2^ = 0%) (Fig. 2D). The area of allodynia (expressed as % of basal value) did not change between the RF and control treatments (SMD: 0.33; 95% CI: −0.21 to 0.88; *P* = 0.23; I^2^ = 7.5%) (Fig. 2E).

#### 3.4.1. Subgroup analyses (primary outcome)

Pain intensity reduction did not persist as significant when studies were separated into those using low RF (≤0.1 μg/kg/min) and intermediate (>0.1 μg/kg/min) doses (see OSC, Appendix 2, Figures I, II, available at http://links.lww.com/PAIN/B959).

The subgroup analysis, including only studies on electrical stimulation, confirmed a significant increase in pain intensity after RF discontinuation (SMD: 0.79, 95% CI: 0.21-1.38; *P* = 0.008, I^2^ = 63%) (OSC, Appendix 2, Figures III, available at http://links.lww.com/PAIN/B959). Conversely, the analysis considering studies on nonelectrical stimulations (heat pain test and cold pain test) showed no significant results (see OSC, Appendix 2, Figures IV, V, available at http://links.lww.com/PAIN/B959).

#### 3.4.2. Subgroup analyses (secondary outcomes)

The increase of hyperalgesia area persisted even if studies were distinguished in low (SMD: 0.42, 95% CI: 0.07-0.76; *P* = 0.02, I^2^ = 0%) and intermediate RF doses (SMD: 0.94, 95% CI: 0.30-1.57; *P* = 0.004, I^2^ = 13%) (see OSC, Appendix 3, Figures VI, VII, available at http://links.lww.com/PAIN/B959).

An insufficient number of studies were available to perform subgroup analyses for allodynia.

### 3.5 Sensitivity analyses on primary outcome

Sensitivity analysis including only studies performed with TCI showed a significant increase in pain intensity after RF withdrawal with no heterogeneity (SMD: 0.61, 95% CI: 0.16-1.05; *P* = 0.007, I^2^ = 0%). On the contrary, the analysis including studies performed without TCI did not show any significant result (see OSC, Appendix 4, Figures VIII, IX, available at http://links.lww.com/PAIN/B959).

The sensitivity analysis on sex differences was not performed as the percentage of male subjects was too high (93.4%).

Sensitivity analysis including studies in which the duration of RF infusion was 30 minutes confirmed the greater pain intensity after RF withdrawal with a similar heterogeneity (SMD: 0.76, 95% CI: 0.23-1.29; *P* = 0.005, I^2^ = 66%). Meanwhile, the analysis including studies with RF infusion duration >30 minutes was not significant (see OSC, Appendix 4, Figures X, XI, available at http://links.lww.com/PAIN/B959).

### 3.6. Risk of bias and Grading of Recommendations, Assessment, Development, and Evaluations assessment

**Figure 3.**
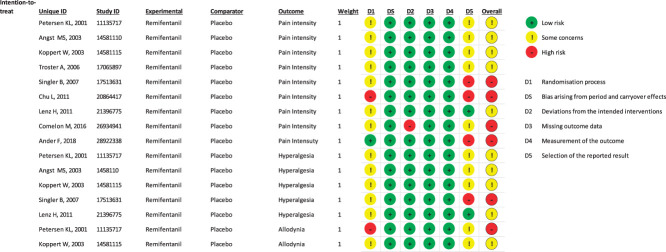
Review authors' judgment of each of the risk-of-bias items across the included studies evaluated using the Cochrane Risk of Bias 2 tool (Rob 2).

**Figure 4.**
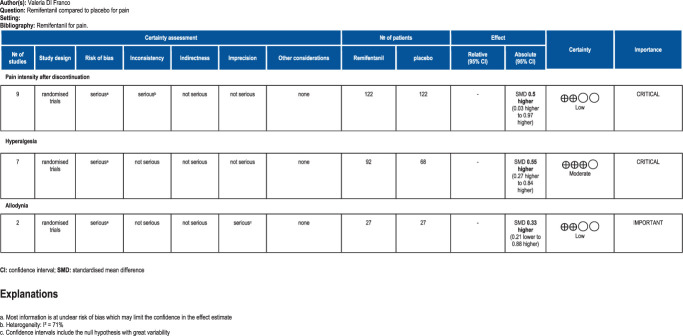
Certainty of evidence assessment for the randomised controlled trials with cross-over design included in the meta-analysis.

The assessment of the methodological quality of included studies is reported in Figure 3 (and in OSC, Appendix 5, available at http://links.lww.com/PAIN/B959). The overall risk for many studies was in the middle category, labeled as “some concerns.” The concerns were typically related to the randomization process^8,17,29,31,38,43,47^ and the selection of the reported results.^8,17,29,38,47^ All studies were double blind, except one^6^ in which it was specifically stated that only the participants were blinded. However, most studies did not detail the blinding process leading to the risk of functional unblinding due to saline solution control. Regarding the primary outcome, 4 studies^6,16,17,43^ showed an overall high risk of bias, due to the randomization process,^16^ selection of reported results,^6,16,43^ or deviation from the intended interventions^17^ (Fig. 3, and OSC, Appendix 5, available at http://links.lww.com/PAIN/B959). All trials had a low risk of bias for bias arising from the washout period and subsequent carry over effect, missing data, and outcome measurement (Fig. 3). It was uncommon that the participants did not adhere to the assigned treatment. Only in one study, 3 HVs were excluded from the analysis for side effects, data error, and compliance problems.^17^ In one study,^16^ some of the details of randomization or random sequence generation and allocation concealment were unclear. Last, tests for the funnel plot asymmetry were not used as fewer than 10 studies were included in the meta-analysis. The certainty of evidence ranged from moderate to low (Fig. 4).

## 4. Discussion

This was the first systematic review, based on 9 RCTs with cross-over design (all published from 2001 onwards) including 122 HVs, aimed at investigating the occurrence of OIH after RF withdrawal in a nonclinical setting. This evidence-based review revealed that, even if RF withdrawal was associated with higher absolute pain scores, no changes were detected compared with baseline values. Interestingly, when analysed separately, we found reduced pain scores in the placebo treatment after withdrawal—compared with baseline—whereas this effect did not occur in the RF treatment. To interpret the findings of pre–post analyses, we should consider that SMDs for postwithdrawal pain intensity suggested small differences between treatments to an extent of being considered insignificant if applied to any clinical setting and research arena. These considerations arise from a previous analysis of the clinical significance of differences in pain intensity. Indeed, for patients with moderate pain, a decrease of 1.3 units for NRS corresponds to a minimal improvement and a meaningful change in pain intensity rises as the severity of baseline pain increases.^15^ Reduction of pain scores in the placebo treatment was mainly observed in studies that used intradermal electrical stimulation.^16,29,31,43,47^ In this human experimental model of pain, the electrical stimulation promotes the activation of a mechano-insensitive class of C no-ciceptors, which is followed by the induction of stable areas of secondary hyperalgesia and allodynia. The stimulation current is gradually increased within the first 15 minutes to target a pain rating of 5 to 6 on an 11-point NRS, but the pain rating tends to be reduced during ongoing electrical stimulation. The pain score did not change from baseline in the RF treatment, despite the development of a significant area of hyperalgesia, likely because the pain threshold decreases from baseline after opioid withdrawal accordingly to previous preclinical and clinical studies performing QST.^46^ Therefore, findings suggest that RF-induced analgesic hypersensitivity seems to have little influence on pain perception in HVs. Clearly, in clinical practice, we need to consider that the degree of pain perception may be also affected by other variables, including demographic, lifestyle, genetic, and psychological factors or specific characteristics of a certain surgical population.^3,32^ Pain perception may be biased by patients' expectations, previous pain experiences, and placebo or nocebo conditioning, with already little known mechanisms.^10^ For this reason, the detection of OIH is very difficult to perform even if it has been overcome by implementing a balanced multimodal analgesic regimen with opioids limited to the minimum necessary for the shortest period possible.^4,36^

Our results do not align with a meta-analysis of RCT in which intraoperative RF infusion is associated with a small but significant increase in acute pain after surgery.^21^ The occurrence of OIH in humans has been previously advocated in patients undergoing surgery and in HVs.^20^ Preclinical and clinical data generally support the development of OIH after RF withdrawal in specific settings, such as acute postsurgical pain ones, when RF was infused at ≥ 0.1 μg/kg/min either alone or with inhalation anaesthetics.^7,12,27^ In particular, previous clinical studies have underlined the development of hyperalgesia after high-dose RF anesthesia^21^; this dose-dependent phenomenon was not fully explored by our meta-analysis as high doses of RF (eg, ≥0.3 μg/kg/min) are not used in HVs likely due to possible respiratory side effects. Therefore, in these conditions, clinicians need to be cautious about the possible occurrence of acute opioid tolerance^24,25,48^ and OIH, which may impair pain treatment or even worsen pre-existing pain. According to the previously reported results, coadministration of anaesthetic drugs, such as propofol,^43,45^ nitrous oxide,^49^ magnesium,^42^ ketamine,^5,39^ and dexmedetomidine,^50^ seem to be helpful to modulate the development of OIH, even if their modulatory effect needs to be further explored. However, there is a lack of validated approaches to diagnose OIH, which may limit the relevance of these results.^9^

For what concerns hyperalgesia, we found that RF infusion induces an enlargement of the area of hyperalgesia after withdrawal compared with basal values, which seems mainly linked to a reduced pain threshold. Some investigators observed a significant enlargement of the area of mechanical hyperalgesia induced by transdermal electrical stimulation after exposure to RF in HVs, and this effect was not dose dependent.^8,16,29,31^ These data are in line with previous studies on animal models, showing that even acute exposures to opioids may induce a decrease in pain threshold and hyperalgesia.^30^ In preclinical studies as well as in HV research investigating OIH, RF is the most extensively studied opioid. However, there are also data suggesting a common hyperalgesic phenomenon for all opioids.^30^ The impact of RF on allodynia was not as clear in our analysis due to limited data.

Regarding possible pathogenic mechanisms underlying OIH, it seems that both peripheral and central mechanisms of neuronal sensitization are involved.^41^ In addition, a crucial role of glial cell activation, particularly microglia, has also been hypothesized in promoting neuroinflammation and sustaining neuronal sensitization. In a preliminary study on the human microglial C20 cell line,^13^ we showed a potential pronociceptive action of RF, through brain-derived neurotrophic factor secretion pathway.^14^ However, when we further expanded these initial observations, focusing on proinflammatory mediators, we demonstrated that RF, at clinically relevant concentrations, is not able to directly modulate the immune activation of human microglia.^19^

This meta-analysis is burdened by several limitations. First, the methodology was heterogeneous among the studies, and the number of patients included did not reach the optimal size leading to type II error. Regarding QST modalities, the lack of difference in RF impact on nociception based on the type of stimulus (ie, electrical vs thermal) was likely due to the low number of included studies. However, it should be noted that electrical stimulation was the only QST modality with a quite standardized protocol across the different studies. Second, the results are driven by RCTs with cross-over design in most cases without an explicit description of the blinding process, introducing the risk of functional unblinding. Notably, we found a RCT with a parallel group design that confirmed increased pain perception during a repetitive cold test in HVs treated with RF in a model of transcranial direct current stimulation,^11^ in line with the results of our meta-analysis. Third, in the studies included in our systematic review, only 6.6% of the total patient population was female individuals. Even if women have a known higher pain threshold,^18^ this issue probably did not affect our findings as the comparability of the cohorts was achieved in most studies. Finally, the heterogeneity of the data we collected was often high (I^2^ > 50%) probably because of the variety of pain models and protocols of RF administration. It should be noted that studies were performed using different sensory modalities to elicit hyperalgesia, such as transdermal electrical stimulation,^16,29,31,43,47^ cold pressor pain,^6,17^ and heat pain test.^8,17,38^ Moreover, washout time varied from 3 days up to 2 weeks among session trials and the infusion time of RF ranged from 30 to 90 minutes. The rate of RF infusion also varied among the studies between 0.05 mcg/kg/min to 0.2 mcg/kg/min.

For the primary outcome, heterogeneity was lowered, including only studies using TCI models that allow keeping the effect site or plasmatic concentrations constant, to the detriment of variable rates during RF infusion, which may impair comparisons with CI modality. Different infusion modalities can influence the development of acute tolerance and hyperalgesia, although they have an equipotential effect on pain control. Interestingly, Richebé et al.^40^ demonstrated that using the TCI model, compared with the CI mode, can decrease hyperalgesia in the early postoperative period after cardiac surgery. The conclusion of the study was supported by the difference in intraoperative infused total RF dose, which was greater in the CI than in the TCI group.^34^ In this regard, the role of abrupt RF withdrawal is a clinically interesting but poorly explored issue.^26^ Unfortunately, in our analysis, all included studies used this modality and only Comelon et al.^17^ demonstrated that heat-induced pain scores were higher than baseline after abrupt RF withdrawal and not after gradual withdrawal concluding that the gradual RF withdrawal may protect from the development of OIH.

Finally, our meta-analysis was essentially based on small trials (sample size ranging from 9 to 19 HVs). Moreover, even if our sensitivity analyses clearly showed that trials with low risks of biases strengthened our results, our quality assessment, performed using the newest and strict RoB 2 tool,^44^ found that publication biases might lead to an overestimation of OIH.

In conclusion, RF induces a mild degree of hyperalgesia in HVs, likely linked to a reduced pain threshold. However, the increased pain perception does not reach enough clinical relevance requiring prevention. If applied to clinical practice, these findings, due to the limited confidence in the effect estimate, do not suggest avoiding RF to overcome the concern for the development of OIH. We do believe that the findings of this systematic review and meta-analysis on HVs would help to understand the mechanisms of hyperalgesia and thus design effective interventions for pain patients.

## Conflict of interest statement

V.D.F. is an employee of Angelini Pharma since April 2023. The other authors declare no conflict of interest.

## Appendix A. Supplemental digital content

Supplemental digital content associated with this article can be found online at http://links.lww.com/PAIN/B959.

## Supplementary Material
