# Supplementary material for: Remifentanil-induced hyperalgesia in healthy volunteers: a systematic review and meta-analysis of randomized controlled trials
Source: Pain. 2023 Nov 30;165(5):972–82. doi: 10.1097/j.pain.0000000000003119 (PMC11017745; doi:10.1097/j.pain.0000000000003119)
Supplement: Supplementary file 1 [file jop-165-0972-s001.pdf]

Online Supplementary Content

**Appendix 1**	**Search Strategy**	Page 2
**Appendix 2**	**Subgroup analyses for pain intensity**	Page 3
**Figure I**	Forest plot for low doses of Remifentanil (infusion rate ≤ 0.1 mcg/kg/min)	Page 3
**Figure II**	Forest plot for intermediate doses of Remifentanil infusion rate (> 0.1 mcg/kg/min)	Page 3
**Figure III**	Forest plot including studies using electrical stimulation model	Page 4
**Figure IV**	Forest plot including studies using heat pain test	Page 4
**Figure V**	Forest plot including studies using cold pressure test	Page 4
**Appendix 3**	**Subgroup analyses for area (expressed as % of basal value) of hyperalgesia**	Page 5
**Figure VI**	Forest plot for low doses of Remifentanil (infusion rate ≤ 0.1 mcg/kg/min)	Page 5
**Figure VII**	Forest plot for intermediate doses of Remifentanil (infusion rate > 0.1 mcg/kg/min)	Page 5
**Appendix 3**	**Sensitivity analyses for pain intensity**	Page 6
**Figure VIII**	Forest plot for target-controlled infusion (TCI)	Page 6
**Figure IX**	Forest plot for controlled infusion (CI)	Page 6
**Figure X**	Forest plot including studies with infusion duration of 30 minutes	Page 7
**Figure XI**	Forest plot including studies with infusion duration >30 minutes	Page 7
**Appendix 5**	**Summary of risk of bias**	Page 8

Appendix 1

Search Strategy remifentanil and hyperalgesia

1. **Cochrane CENTRAL search strategy trough Cochrane Library (27th January 2023) (https://www.cochranelibrary.com/advanced-search/mesh)**

Remifenanil AND hyperalgesia
247 trials

2. Pubmed/MEDLINE search strategy
MEDLINE 2003 to 27h January 2023.

(remifentanil) AND (Hyperalgesia)
"remifentanil"[MeSH Terms] OR "remifentanil"[All Fields] OR "remifentanil s"[All Fields] AND "hyperalgesia"[MeSH Terms] OR "hyperalgesia"[All Fields] OR "hyperalgesias"[All Fields]
290 papers

3. EMBASE search strategy
Database: Embase, up to 27th January 2023.

('remifentanil'/exp OR remifentanil) AND ('hyperalgesia'/exp OR hyperalgesia)
594 hits

4. Scopus Database, up to 27th January 2023
(remifentanil) AND (hyperalgesia
540 papers

Grey literature

- **Google Scholar**: we searched using the following key words
(remifentanil) AND (hyperalgesia)

- **ClinicalTrials.gov**: we searched using the following key words
(remifentanil) AND (hyperalgesia)

Appendix 2. Forest plots of subgroup analyses for pain intensity

Figure I. Forest plot for low doses of Remifentanil (infusion rate ≤ 0.1 mcg/kg/min)

The results show a non-significant effect size favoring the control condition compared to Remifentanil. The effect size is reported as standardized mean difference (SMD) with the corresponding 95% confidence interval (95% CI).

Study or Subgroup	Std. Mean Difference	SE	Weight	Std. Mean Difference IV, Random, 95% CI
Angst 2003	-0.6772	0.4629	19.1%	-0.68 [-1.58, 0.23]
Koppert 2003	1.0678	0.4239	19.9%	1.07 [0.24, 1.90]
Petersen 2001	0.3063	0.3805	20.8%	0.31 [-0.44, 1.05]
Singler 2007	1.934	0.4529	19.3%	1.93 [1.05, 2.82]
Troster 2006	0.3355	0.3681	21.0%	0.34 [-0.39, 1.06]
**Total (95% CI)**			**100.0%**	**0.59 [-0.19, 1.37]**

Heterogeneity: $Tau^2 = 0.63$; $Chi^2 = 18.59$, df = 4 (P = 0.0009); $I^2 = 78\%$
Test for overall effect: Z = 1.47 (P = 0.14)

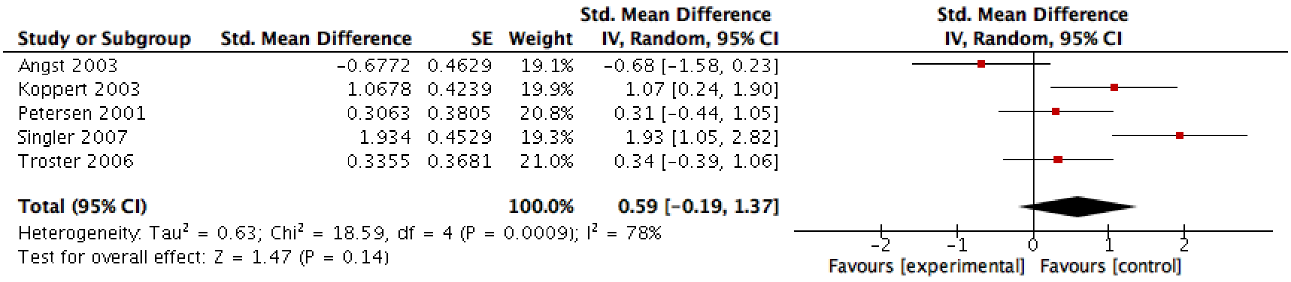

Figure II. Forest plot for intermediate doses of Remifentanil infusion rate (> 0.1 mcg/kg/min)

The results show a non-significant effect size favoring the control condition compared to Remifentanil. The effect size is reported as standardized mean difference (SMD) with the corresponding 95% confidence interval (95% CI).

Study or Subgroup	Std. Mean Difference	SE	Weight	Std. Mean Difference IV, Random, 95% CI
Ander 2018	-0.1383	0.3785	25.9%	-0.14 [-0.88, 0.60]
Chu 2011	0.1235	0.4477	20.8%	0.12 [-0.75, 1.00]
Comelon 2016	0.9358	0.375	26.2%	0.94 [0.20, 1.67]
Lenz 2011	0.6118	0.3629	27.2%	0.61 [-0.10, 1.32]
**Total (95% CI)**			**100.0%**	**0.40 [-0.08, 0.88]**

Heterogeneity: $Tau^2 = 0.09$; $Chi^2 = 4.78$, df = 3 (P = 0.19); $I^2 = 37\%$
Test for overall effect: Z = 1.63 (P = 0.10)

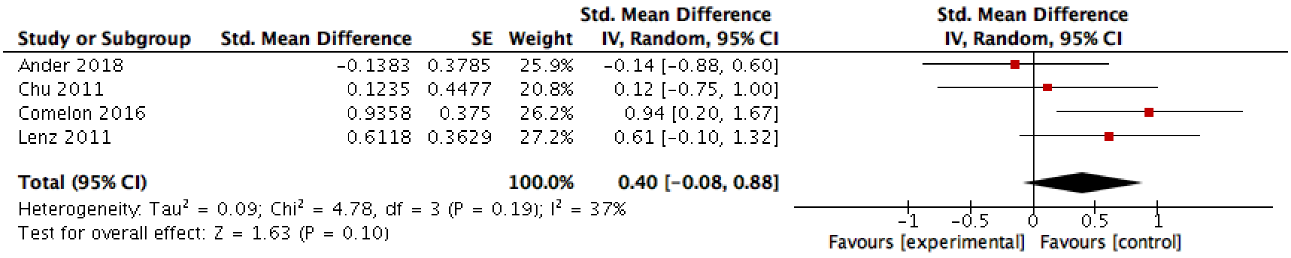

Figure III. Forest plot including studies using electrical stimulation model

The results show a significant effect size favoring the control condition compared to Remifentanil. The effect size is reported as standardized mean difference (SMD) with the corresponding 95% confidence interval (95% CI).

Study or Subgroup	Std. Mean Difference	SE	Weight	Std. Mean Difference IV, Random, 95% CI
Chu 2011	0.1235	0.4477	18.7%	0.12 [−0.75, 1.00]
Koppert 2003	1.0678	0.4239	19.5%	1.07 [0.24, 1.90]
Lenz 2011	0.6118	0.3629	21.8%	0.61 [−0.10, 1.32]
Singler 2007	1.934	0.4529	18.5%	1.93 [1.05, 2.82]
Troster 2006	0.3355	0.3681	21.6%	0.34 [−0.39, 1.06]
**Total (95% CI)**			**100.0%**	**0.79 [0.21, 1.38]**

Heterogeneity: $Tau^2 = 0.28$; $Chi^2 = 10.76$, df = 4 (P = 0.03); $I^2 = 63\%$
Test for overall effect: Z = 2.65 (P = 0.008)

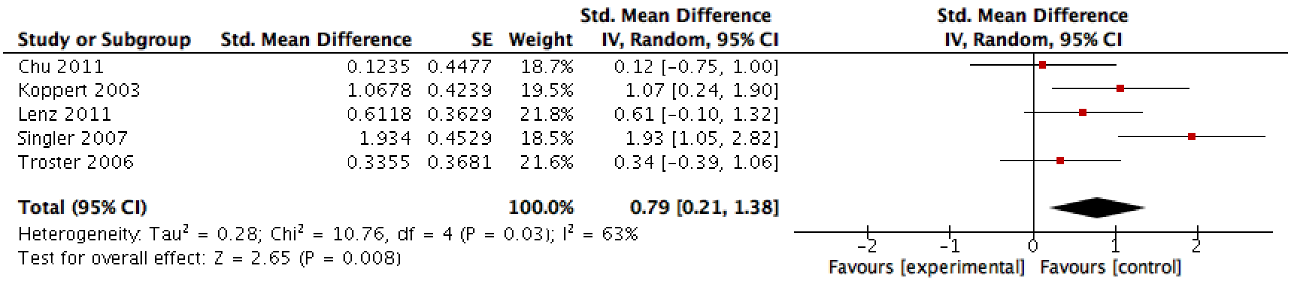

Figure IV. Forest plot including studies using heat pain test

The results show a non-significant effect size favoring the control condition compared to Remifentanil. The effect size is reported as standardized mean difference (SMD) with the corresponding 95% confidence interval (95% CI).

Study or Subgroup	Std. Mean Difference	SE	Weight	Std. Mean Difference IV, Random, 95% CI
Angst 2003	−0.6772	0.4629	30.8%	−0.68 [−1.58, 0.23]
Comelon 2016	0.9358	0.375	34.7%	0.94 [0.20, 1.67]
Petersen 2001	0.3063	0.3805	34.5%	0.31 [−0.44, 1.05]
**Total (95% CI)**			**100.0%**	**0.22 [−0.65, 1.10]**

Heterogeneity: $Tau^2 = 0.43$; $Chi^2 = 7.33$, df = 2 (P = 0.03); $I^2 = 73\%$
Test for overall effect: Z = 0.50 (P = 0.62)

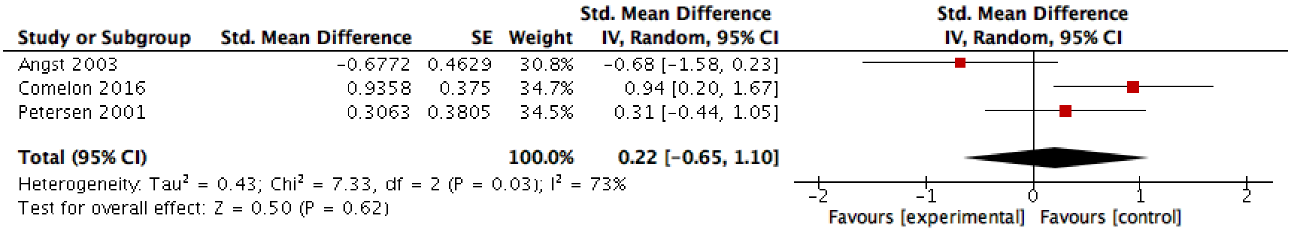

Figure V. Forest plot including studies using cold pressure test

The results show a non-significant effect size favoring the control condition compared to Remifentanil. The effect size is reported as standardized mean difference (SMD) with the corresponding 95% confidence interval (95% CI).

Study or Subgroup	Std. Mean Difference	SE	Weight	Std. Mean Difference IV, Random, 95% CI
Ander 2018	−0.1383	0.3785	50.3%	−0.14 [−0.88, 0.60]
Comelon 2016	1.3452	0.3965	49.7%	1.35 [0.57, 2.12]
**Total (95% CI)**			**100.0%**	**0.60 [−0.86, 2.05]**

Heterogeneity: $Tau^2 = 0.95$; $Chi^2 = 7.32$, df = 1 (P = 0.007); $I^2 = 86\%$
Test for overall effect: Z = 0.81 (P = 0.42)

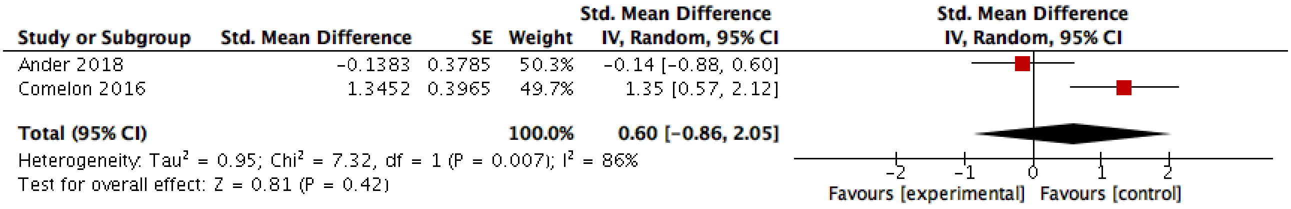

Appendix 3. Forest plots of subgroup analyses for hyperalgesia

Figure VI. Forest plot for low doses of Remifentanil (infusion rate ≤ 0.1 mcg/kg/min)

The results show a significant effect size favoring the control condition compared to Remifentanil. The effect size is reported as standardized mean difference (SMD) with the corresponding 95% confidence interval (95% CI).

Study or Subgroup	Std. Mean Difference	SE	Weight	Std. Mean Difference IV, Random, 95% CI
Angst 2003	0.8146	0.4697	14.0%	0.81 [−0.11, 1.74]
Koppert 2003	0.4567	0.3982	19.5%	0.46 [−0.32, 1.24]
Petersen 2001	0.7048	0.3914	20.2%	0.70 [−0.06, 1.47]
Singler 2007	0.1444	0.3657	23.1%	0.14 [−0.57, 0.86]
Troster 2006	0.1679	0.3659	23.1%	0.17 [−0.55, 0.89]
**Total (95% CI)**			**100.0%**	**0.42 [0.07, 0.76]**

Heterogeneity: $Tau^2 = 0.00$; $Chi^2 = 2.29$, df = 4 (P = 0.68); $I^2 = 0\%$
Test for overall effect: Z = 2.38 (P = 0.02)

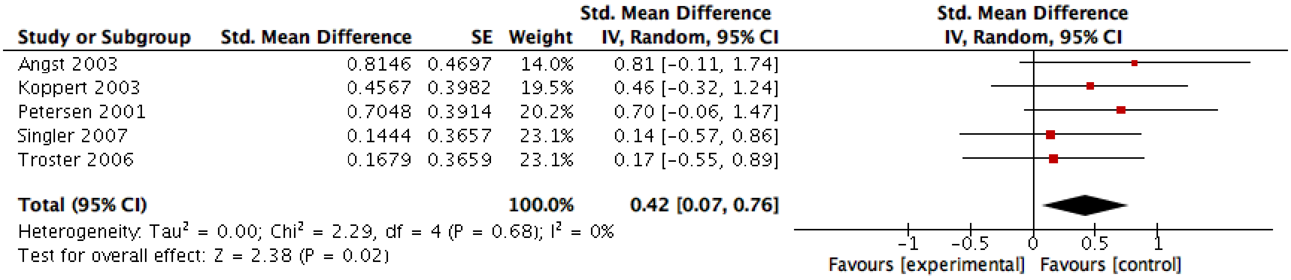

Figure VII. Forest plot for intermediate doses of Remifentanil (infusion rate > 0.1 mcg/kg/min)

The results show a significant effect size favoring the control condition compared to Remifentanil. The effect size is reported as standardized mean difference (SMD) with the corresponding 95% confidence interval (95% CI).

Study or Subgroup	Std. Mean Difference	SE	Weight	Std. Mean Difference IV, Random, 95% CI
Chu 2011	1.3632	0.5078	36.2%	1.36 [0.37, 2.36]
Lenz 2011	0.6922	0.3654	63.8%	0.69 [−0.02, 1.41]
**Total (95% CI)**			**100.0%**	**0.94 [0.30, 1.57]**

Heterogeneity: $Tau^2 = 0.03$; $Chi^2 = 1.15$, df = 1 (P = 0.28); $I^2 = 13\%$
Test for overall effect: Z = 2.90 (P = 0.004)

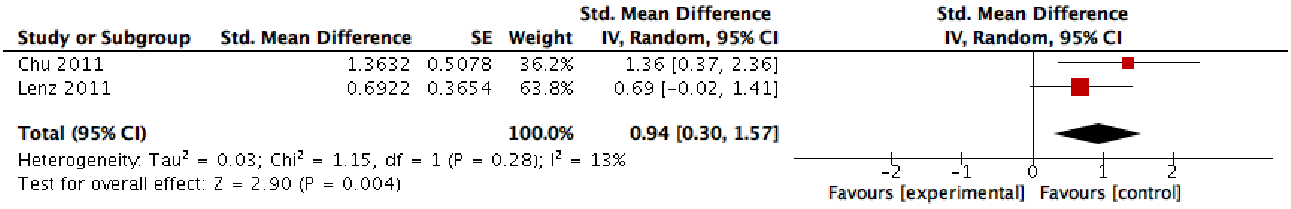

Appendix 4. Forest plots of sensitivity analyses for pain intensity

Figure VIII. Forest plot for target-controlled infusion (TCI)

The results show a significant effect size favoring the control condition compared to Remifentanil. The effect size is reported as standardized mean difference (SMD) with the corresponding 95% confidence interval (95% CI).

Study or Subgroup	Std. Mean Difference	SE	Weight	Std. Mean Difference IV, Random, 95% CI
Chu 2011	0.1235	0.4477	25.3%	0.12 [-0.75, 1.00]
Comelon 2016	0.9358	0.375	36.1%	0.94 [0.20, 1.67]
Lenz 2011	0.6118	0.3629	38.6%	0.61 [-0.10, 1.32]
**Total (95% CI)**			**100.0%**	**0.61 [0.16, 1.05]**

Heterogeneity: $Tau^2 = 0.00$; $Chi^2 = 1.94$, df = 2 (P = 0.38); $I^2 = 0\%$
Test for overall effect: Z = 2.69 (P = 0.007)

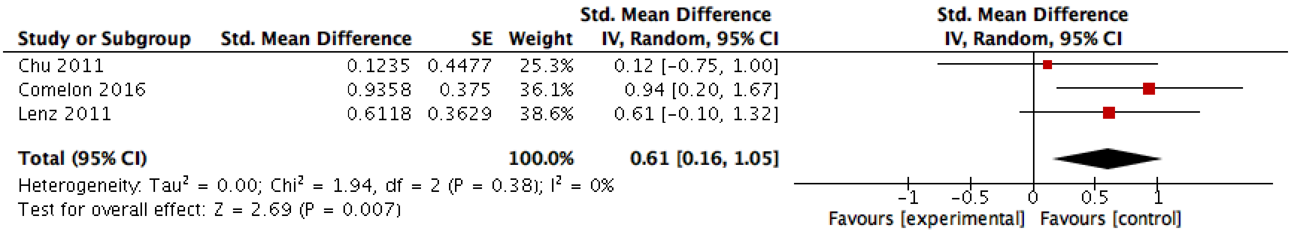

Figure IX. Forest plot for controlled infusion (CI)

The results show a non-significant effect size favoring the control condition compared to Remifentanil. The effect size is reported as standardized mean difference (SMD) with the corresponding 95% confidence interval (95% CI).

Study or Subgroup	Std. Mean Difference	SE	Weight	Std. Mean Difference IV, Random, 95% CI
Ander 2018	-0.1383	0.3785	17.3%	-0.14 [-0.88, 0.60]
Angst 2003	-0.6772	0.4629	15.7%	-0.68 [-1.58, 0.23]
Koppert 2003	1.0678	0.4239	16.4%	1.07 [0.24, 1.90]
Petersen 2001	0.3063	0.3805	17.3%	0.31 [-0.44, 1.05]
Singler 2007	1.934	0.4529	15.9%	1.93 [1.05, 2.82]
Troster 2006	0.3355	0.3681	17.5%	0.34 [-0.39, 1.06]
**Total (95% CI)**			**100.0%**	**0.46 [-0.21, 1.14]**

Heterogeneity: $Tau^2 = 0.55$; $Chi^2 = 21.44$, df = 5 (P = 0.0007); $I^2 = 77\%$
Test for overall effect: Z = 1.34 (P = 0.18)

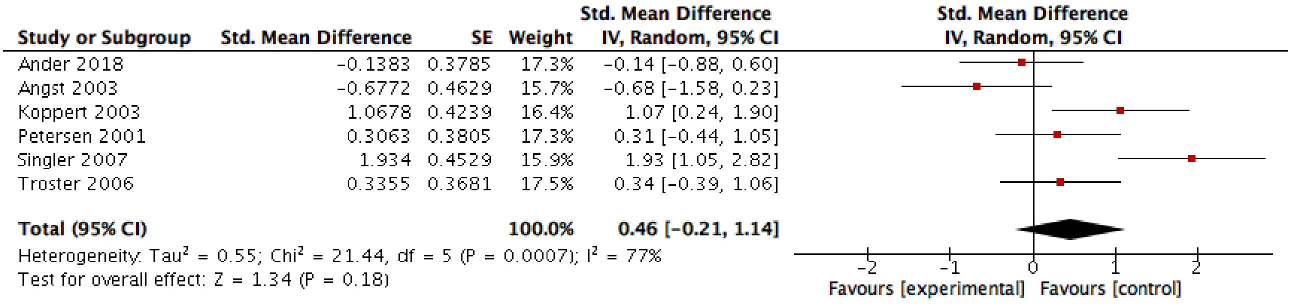

Figure X. Forest plot including studies with infusion duration of 30 minutes

The results show a significant effect size favoring the control condition compared to Remifentanil. The effect size is reported as standardized mean difference (SMD) with the corresponding 95% confidence interval (95% CI).

Study or Subgroup	Std. Mean Difference	SE	Weight	Std. Mean Difference IV, Random, 95% CI
Ander 2018	-0.1383	0.3785	17.1%	-0.14 [-0.88, 0.60]
Comelon 2016	0.9358	0.375	17.2%	0.94 [0.20, 1.67]
Koppert 2003	1.0678	0.4239	15.8%	1.07 [0.24, 1.90]
Lenz 2011	0.6118	0.3629	17.6%	0.61 [-0.10, 1.32]
Singler 2007	1.934	0.4529	15.0%	1.93 [1.05, 2.82]
Troster 2006	0.3355	0.3681	17.4%	0.34 [-0.39, 1.06]
**Total (95% CI)**			**100.0%**	**0.76 [0.23, 1.29]**

Heterogeneity: $Tau^2 = 0.29$; $Chi^2 = 14.51$, df = 5 (P = 0.01); $I^2 = 66\%$
Test for overall effect: Z = 2.80 (P = 0.005)

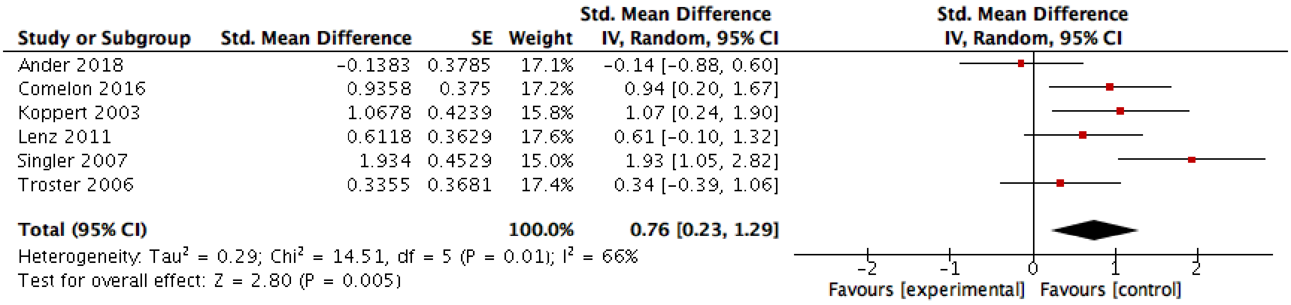

Figure XI. Forest plot including studies with infusion duration >30 minutes

The results show a non-significant effect size favoring the control condition compared to Remifentanil. The effect size is reported as standardized mean difference (SMD) with the corresponding 95% confidence interval (95% CI).

Study or Subgroup	Std. Mean Difference	SE	Weight	Std. Mean Difference IV, Random, 95% CI
Angst 2003	-0.6772	0.4629	29.8%	-0.68 [-1.58, 0.23]
Chu 2011	0.1235	0.4477	31.2%	0.12 [-0.75, 1.00]
Petersen 2001	0.3063	0.3805	39.0%	0.31 [-0.44, 1.05]
**Total (95% CI)**			**100.0%**	**-0.04 [-0.62, 0.54]**

Heterogeneity: $Tau^2 = 0.08$; $Chi^2 = 2.85$, df = 2 (P = 0.24); $I^2 = 30\%$
Test for overall effect: Z = 0.15 (P = 0.88)

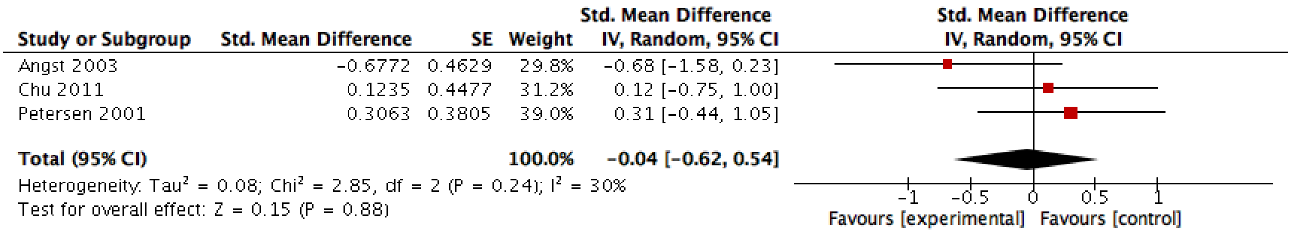

Appendix 5. Summary of risk of bias

Quality assessment of the studies included in the systematic review and meta-analysis was performed using Version 2 of the Cochrane risk-of-bias tool for randomized trials (RoB 2). Risk of bias was independently assessed by two review team members (CDR, VDF). Any conflicts were discussed with a third author (PA or FR).

First Author / Year of publication	PubMed ID	Experimental	Comparator	Outcome	Overall Bias	Comments
Petersen KL / 2001	11135717	RF	Placebo	Pain Intensity	Some Concerns	The randomization procedure is not described. Information about allocation concealment is not provided. One single value for baseline is provided for pain intensity. The study was carried out under double-blind conditions. Analysis was performed on an ITT basis. Data were presented as graphs. The intensity of pain perception was evaluated using a visual analog pain intensity scale. The same method of data collection was used in both sessions. The study was carried out under double-blind conditions. The authors did not indicate if they used a pre-specified analysis plan and did not specify if the data analysis was carried out under blind conditions. Only one outcome measurement was used. Only one analysis was done. Trial sessions were 1 week apart; thus, no carryover effect was present.
Angst MS / 2003	14581110	RF	Placebo	Pain Intensity	Some Concerns	Subjects were randomly allocated. The randomization procedure is not described. Information about allocation concealment is not provided. Baseline values for pain intensity were similar. The study was carried out under double blind conditions. Study volunteers and investigators performing the pain tests were blinded to treatment. Analysis was performed on an ITT basis. All patients completed the study. The intensity of pain perception was evaluated using a visual analog pain intensity scale. The same method of data collection was used in the two sessions. The study was carried out under double blind conditions. The authors did not indicate if they used a pre-specified analysis plan and did not specify if the data analysis was carried out under blind conditions. One scale was used for outcome measurements. All time-points are reported. One analysis was carried out. Trial sessions were at least 3 days apart; thus, no carryover is expected.
Koppert W / 2003	14581115	RF	Placebo	Pain Intensity	Some Concerns	Subjects were randomly allocated. The randomization procedure is not described. Information about allocation concealment is not provided. Baseline values for pain intensity were similar. The study was carried out in double blind conditions. Analysis was performed on an ITT basis. Data were presented as graphs. The

						intensity of pain perception was evaluated using a numerical rating scale (NRS). The same method of data collection was used in the two sessions. The study was carried out under double blind conditions. The authors did not indicate if they used a pre-specified analysis plan and did not specify if the data analysis was carried out under blind conditions. One scale was used for outcome measurements. All time-points are reported. One type of analysis was carried out. Trial sessions were at least 1 week apart; thus, no carryover is expected.
Troster A / 2006	17065897	RF	Placebo	Pain Intensity	Some Concerns	Subjects were randomly allocated. The randomization procedure is not described. Information about allocation concealment is not provided. No relevant differences between the treatment groups were observed at baseline. The study was carried out in double-blind conditions. Analysis was performed on an ITT basis. Data are expressed as mean +/- SEM of n=15, thus suggesting that the outcome was available for all subjects. The intensity of pain perception was evaluated using a numerical rating scale (NRS). The authors used the same method of data collection for the different intervention sessions. The study was carried out under double-blind conditions. The authors did not indicate if they used a pre-specified analysis plan and did not specify if the data analysis was carried out under blind conditions. One scale was used for outcome measurements. All time-points are reported. Treatment effects over time were evaluated using two-way repeated measures analysis of variance (ANOVA) including the effects “treatment” and “time course.” Post hoc testing was performed as one analysis was carried out. Trial sessions were 2 weeks apart.
Singler B / 2007	17513631	RF	Placebo	Pain Intensity	High	Subjects were randomly allocated. The randomization procedure is not described. Information about allocation concealment is not provided. No relevant session differences were observed. The study was carried out under double-blind conditions. We can hypothesize that all subjects were included in the analysis. Data were presented as graphs. The intensity of pain perception was evaluated using a numerical rating scale (NRS). The authors used the same method of data collection for the different intervention sessions. The study was carried out under double-blind conditions. The authors did not indicate if they used a pre-specified analysis plan and did not specify if the data analysis was carried out under blind conditions. One scale was used for outcome measurements. All time-points are reported. If significant treatment effects were detected, additional analysis was performed. Trial sessions were at least 2 weeks apart.
Chu LF / 2011	20864417	RF	Placebo	Pain Intensity	High	Subjects were randomly allocated. The randomization procedure is not described. Information about allocation concealment is not provided. Data suggest differences in the baseline among the two sessions, although no comments by the authors were made. The study was carried out in double-blind conditions. Analysis was performed on an ITT basis. Data were presented as graphs. The intensity of pain perception was evaluated using a visual analog pain intensity scale. The authors used the same method of data collection for the different

						intervention sessions. The study was carried out under double-blind conditions. The authors did not indicate if they used a pre-specified analysis plan and did not specify if the data analysis was carried out under blind conditions. One scale was used for outcome measurements. All time-points are reported. Outcome variables were compared by using a 2-tailed paired parametric or nonparametric test. Trial sessions were at least 5 days apart.
Lenz H / 2011	21396775	RF	Placebo	Pain Intensity	Some concerns	Subjects were randomly allocated. The randomization procedure is not described. Information about allocation concealment is not provided. No relevant differences were found among different groups of treatment at baseline. The study was carried out in double-blind conditions. We can hypothesize that all subjects were included in the analysis. Data were presented as graphs. The intensity of pain perception was evaluated using a numerical rating scale (NRS). The authors used the same method of data collection for the different intervention sessions. The study was carried out under double-blind conditions. Sample size was calculated based on results from a previous study. Based on a pre-specified power analysis, the authors decided to include 16 subjects. One scale was used for outcome measurements. All time-points are reported. A linear mixed-model procedure was used for the analysis. Trial sessions were at least 1 week apart.
Comelon M / 2016	26934941	RF	Placebo	Pain Intensity	High	Quote: "Computer generated codes stored in sequentially numbered envelopes secured randomization of the sessions". Information about allocation sequence concealment is not reported. Baseline values for pain intensity were similar. Quote: "A nurse anaesthetist not participating in the handling or evaluation of the subjects prepared remifentanil and saline in 50 ml syringes for infusion according to the randomization, thus blinding the investigators and the subjects". The analysis was not performed according to the ITT model. 3 patients excluded from the analysis for side effects, data error, compliance problems. Data were presented as graphs. The intensity of pain perception was evaluated using a numerical rating scale (NRS). The authors used the same method of data collection for the different intervention sessions. The study was carried out under double-blind conditions. The authors did not indicate if they used a pre-specified analysis plan and did not specify if the data analysis was carried out under blind conditions. One scale was used for outcome measurements. All time-points are reported. One type of analysis was carried out. There was a minimum interval of 4 days between each session, thus excluding possible carryover effects.
Ander F / 2018	28922338	RF	Placebo	Pain Intensity	High	Quote: "the study participants were randomized to the sequence of intervention using sealed opaque envelopes. Envelopes were prepared by departmental staff who had no other part in the study". Baseline values for pain intensity were similar. Quote: "The study participants were blinded to the intervention sequence". The analysis was performed according to the ITT model. Data for this outcome were available for all 14 participants. The intensity of pain perception was evaluated using a numerical rating scale (NRS). The authors used the same

						method of data collection for the different intervention sessions. The study was carried out under double-blind conditions. The authors did not indicate if they used a pre-specified analysis plan and did not specify if the data analysis was carried out under blind conditions. Only one outcome measurement was used. Multiple eligible analyses were reported. Each session was separated by at least 3 days, thus excluding possible carryover effects.
Angst MS / 2003	1458110	RF	Placebo	Hyperalgesia	Some Concerns	Subjects were randomly allocated. The randomization procedure is not described. Information about allocation concealment is not provided. Quote: "The area of mechanical hyperalgesia to punctuated stimulation before drug administration was not significantly different among the four drug treatments". The study was carried out under double blind conditions. Study volunteers and investigators performing the pain tests were blinded to treatment. Analysis was performed on at ITT basis. All patients completed the study. The mechanical hyperalgesia was evaluated using an area in cm^2. The same method of data collection was used in the two sessions. The study was carried out under double blind conditions. The authors did not indicate if they used a pre-specified analysis plan and did not specify if the data analysis was carried out under blind conditions. One scale was used for outcome measurements. All time-points are reported. One analysis was carried out. Trial sessions were at least 3 days apart; thus, no carryover is expected.
Koppert W / 2003	14581115	RF	Placebo	Hyperalgesia	Some Concerns	Subjects were randomly allocated. The randomization procedure is not described. Information about allocation concealment is not provided. Baseline values for hyperalgesic area were similar. The study was carried out in double blind conditions. Analysis was performed on ITT basis. Data were presented as graphs. The mechanical hyperalgesia was evaluated using an area in cm^2. The same method of data collection was used in the two sessions. The study was carried out under double blind conditions. The authors did not indicate if they used a pre-specified analysis plan and did not specify if the data analysis was carried out under blind conditions. One scale was used for outcome measurements. All time-points are reported. One type of analysis was carried out. Trial sessions were at least 1 week apart; thus, no carryover is expected.
Lenz H / 2011	21396775	RF	Placebo	Hyperalgesia	Some Concerns	Subjects were randomly allocated. The randomization procedure is not described. Information about allocation concealment is not provided. No relevant differences were found among different groups of treatments at baseline. The study was carried out in double-blind conditions. We can hypothesize that all subjects were included in the analysis. Data were presented as graphs. Mechanical hyperalgesia is evaluated using an area in %. The authors used the same method of data collection for the different intervention sessions. The study was carried out under double-blind conditions. Sample size was calculated based on results from a previous study. Based on a pre-specified power analysis, the authors decided to

						include 16 subjects. One scale was used for outcome measurements. All time-points are reported. A linear mixed-model procedure was used for the analysis. Trial sessions were at least 1 week apart.
Singler B / 2007	17513631	RF	Placebo	Hyperalgesia	High	Subjects were randomly allocated. The randomization procedure is not described. Information about allocation concealment is not provided. No relevant session differences were observed. The study was carried out under double-blind conditions. We can hypothesize that all subjects were included in the analysis. Data were presented as graphs. Mechanical hyperalgesia was evaluated using an area in %. The authors used the same method of data collection for the different intervention sessions. The study was carried out under double-blind conditions. The authors did not indicate if they used a pre-specified analysis plan and did not specify if the data analysis was carried out under blind conditions. One scale was used for outcome measurements. All time-points are reported. If significant treatment effects were detected additional analysis was performed. Trial sessions were at least 2 weeks apart.
Petersen KL / 2001	11135717	RF	Placebo	Hyperalgesia	Some Concerns	The randomization procedure is not described. Information about allocation concealment is not provided. The baseline areas of secondary hyperalgesia to von Frey hair stimulation were similar for remifentanil and placebo. The study was carried out under double-blind conditions. Analysis was performed on an ITT basis. Data were presented as graphs and as percentage of baseline. Secondary hyperalgesia to von Frey hair stimulation was evaluated using an area in cm2 and as %. The same method of data collection was used in both sessions. The study was carried out under double-blind conditions. The authors did not indicate if they used a pre-specified analysis plan and did not specify if the data analysis was carried out under blind conditions. Only one outcome measurement was used. Only one analysis was performed. Trial sessions were 1 week apart; thus no carryover effect was present.
Petersen KL / 2001	11135717	RF	Placebo	Allodynia	High	The randomization procedure is not described. Information about allocation concealment is not provided. The baseline areas of secondary hyperalgesia to brush stimulation (allodynia) were slightly smaller on the RF-day than on the placebo day. The study was carried out under double-blind conditions. Analysis was performed on an ITT basis. Data were presented as graphs and percentage of the baseline. The allodynia was evaluated using an area in cm^2 and as %. The same method of data collection was used in both sessions. The study was carried out under double-blind conditions. The authors did not indicate if they used a pre-specified analysis plan and did not specify if the data analysis was carried out under blind conditions. Only one outcome measurement was used. Only one analysis was done. Trial sessions were 1 week apart; thus, no carryover effect was present.

Koppert W / 2003	14581115	RF	Placebo	Allodynia	Some Concerns	Subjects were randomly allocated. The randomization procedure is not described. Information about allocation concealment is not provided. Baseline values for allodynic area were similar. The study was carried out in double blind conditions. Analysis was performed on ITT basis. Data were presented as graphs.<br>The allodynia was evaluated using an area in cm^2. The same method of data collection was used in the 2 sessions. The study was carried out under double blind conditions. The authors did not indicate if they used a pre-specified analysis plan and did not specify if the data analysis was carried out under blind conditions. Outcome was evaluated using just an area in cm2. All time points are reported. One type of analysis was carried out. Trial sessions were at least 1 week apart; thus, no carryover is expected.
